# *BRAF* Mutations Are Associated with Poor Survival Outcomes in Advanced-stage Mismatch Repair-deficient/Microsatellite High Colorectal Cancer

**DOI:** 10.1093/oncolo/oyab055

**Published:** 2022-02-09

**Authors:** Elaine Tan, Junmin Whiting, Hao Xie, Iman Imanirad, Estrella Carballido, Seth Felder, Jessica Frakes, Quanxing Mo, Christine Walko, Jennifer B Permuth, Katelyn Sommerer, Richard Kim, Daniel A Anaya, Jason B Fleming, Ibrahim Halil Sahin

**Affiliations:** Department of Gastrointestinal Oncology, H. Lee Moffitt Cancer Center and Research Institute, Tampa, FL, USA; Department of Biostatistics and Bioinformatics, H. Lee Moffitt Cancer Center and Research Institute, Tampa, FL, USA; Department of Gastrointestinal Oncology, H. Lee Moffitt Cancer Center and Research Institute, Tampa, FL, USA; Department of Gastrointestinal Oncology, H. Lee Moffitt Cancer Center and Research Institute, Tampa, FL, USA; Department of Gastrointestinal Oncology, H. Lee Moffitt Cancer Center and Research Institute, Tampa, FL, USA; Department of Gastrointestinal Oncology, H. Lee Moffitt Cancer Center and Research Institute, Tampa, FL, USA; Department of Gastrointestinal Oncology, H. Lee Moffitt Cancer Center and Research Institute, Tampa, FL, USA; Department of Biostatistics and Bioinformatics, H. Lee Moffitt Cancer Center and Research Institute, Tampa, FL, USA; Department of Individualized Cancer Management, H. Lee Moffitt Cancer Center and Research Institute, Tampa, FL, USA; Department of Gastrointestinal Oncology, H. Lee Moffitt Cancer Center and Research Institute, Tampa, FL, USA; Department of Cancer Epidemiology, H. Lee Moffitt Cancer Center and Research Institute, Tampa, FL, USA; Department of Gastrointestinal Oncology, H. Lee Moffitt Cancer Center and Research Institute, Tampa, FL, USA; Department of Gastrointestinal Oncology, H. Lee Moffitt Cancer Center and Research Institute, Tampa, FL, USA; Department of Gastrointestinal Oncology, H. Lee Moffitt Cancer Center and Research Institute, Tampa, FL, USA; Department of Gastrointestinal Oncology, H. Lee Moffitt Cancer Center and Research Institute, Tampa, FL, USA; Department of Gastrointestinal Oncology, H. Lee Moffitt Cancer Center and Research Institute, Tampa, FL, USA

**Keywords:** colorectal cancer, mismatch repair deficiency, microsatellite instability high, *BRAF* V600E, prognosis, KRAS, NRAS, late-onset disease

## Abstract

**Background:**

Mismatch repair-deficient (MMR-D)/microsatellite instability-high (MSI-H) metastatic colorectal cancer (mCRC) is a unique disease entity with growing interest given the rise of young-onset CRC. Given its heterogeneous behavior and potential for highly effective treatment outcomes, we sought to identify the clinical and molecular features that offer prognostic value for MMR-D CRC.

**Materials/Methods:**

This was a retrospective cohort study of patients with metastatic CRC with MMR-D or microsatellite instability in a real-world database. Overall survival (OS) was determined by the date of metastatic disease to date of death with stratification made based on factors including *BRAF* and *RAS* mutation status, age, and MMR protein loss type.

**Results:**

There were 1101 patients in the study. Patients with *BRAF* mutations had worse OS compared with patients with wild-type *BRAF* with a median survival of 18.9 months versus 33.2 months (hazard ratio [HR] 1.52, 95% confidence interval [CI]: 1.25-1.86, *P* < .001). Patients with age >50 were found to have decreased OS versus age ≤50 with a median survival of 21.4 months versus 38.7 months (HR 1.66, 95% CI: 1.33-2.07, *P* < .001). *BRAF* mutations and age >50 remained significant predictors of OS in multivariate analysis.

**Conclusion:**

*BRAF* mutations and age >50 are associated with worse survival outcomes for patients with MMR-D mCRC. *RAS* mutations and specific MMR alterations are not associated with survival outcomes.

## Teaser

Mismatch repair deficiency (MMR-D) or microsatellite instability-high (MSI-high) disease is prevalent in populations of patients with metastatic colorectal cancer (mCRC). This article reports prognostic factors in MMR-D/MSI-H mCRC in a large patient cohort with a real-world database.

**Implications for Practice**

The results of this study reveal clinically relevant markers that predict overall survival for patients with mismatch repair-deficient metastatic colorectal cancer.

## Introduction

Colorectal cancer (CRC) is one of the most common malignancies with a continually rising incidence in young adults; currently, it is the fourth leading cause of cancer-related death worldwide.^1^ Predicted to be the second leading cause of cancer-related death for people aged 20-49 by 2040, CRC presents a concerning trend in young adults.^2^

More prominent in this population is the prevalence of mismatch repair deficiency (MMR-D) or microsatellite instability-high (MSI-high) disease, in up to 5% of metastatic CRC (mCRC).^3^ MMR-D is characterized by the loss of expression or function of any of the MMR genes including, but not limited to, MLH1, PMS2, MSH2, or MSH6. This functional protein loss in MMR genes leads to impaired repair of mismatch nucleotides that occurs during DNA replication. Repeated sequences of DNA, known as microsatellites, are particularly susceptible to errors in cases of MMR-D; accumulation of these errors in these DNA regions then leads to variable sizes of microsatellites called microsatellite instability (MSI). This subsequently leads to frameshift mutations and a high tumor mutation burden.^4^

MMR deficiency is classified as either being secondary to germline mutations versus sporadic mutations, which include but not limited to those with *BRAF* V600E mutation with MLH1 promoter hypermethylation. Making this distinction is important given the various implications: patients with sporadic MMR-D tend to be older, have poorly differentiated disease, and have less sensitivity to chemotherapy.^5^

Translation of genes with frameshift mutation alterations leads to the formation of mutation-associated neoantigens (MANAs), which is a landmark feature of MMR-D/MSI-H tumors. These MANAs are recognized by MANA-specific T cells to subsequently evoke an anti-tumor immune response orchestrated by T cells. Notably, tumor-infiltrating lymphocytes (TILs) are increased in MSI-H/MMR-D tumors, compared with microsatellite stable (MSS) tumors.^6^ The MANA-mediated antitumor immune response in MMR-D/MSI-H tumors observed in preclinical studies triggered clinical trials with immune checkpoint inhibitors and led to practice-changing studies in the last decade. For example, KEYNOTE-177 demonstrated a significant improvement in median progression-free survival (PFS) for patients with MMR-D/MSI-H advanced CRC treated with pembrolizumab at 16.5 months versus 8.2 months with chemotherapy (hazard ratio [HR] 0.60, 95%: confidence interval [CI], 0.45-0.80, *P* = .0002), establishing pembrolizumab as a standard of care first-line option for this population.^7^

The majority of clinical trials have studied patients with MMR-D/MSI-H disease collectively. However, various clinical and molecular features, other than being characterized as MMR-D/MSI-H, may lead to differences in clinical behavior and response to therapies. For example, the *BRAF* V600E mutation and specific MMR gene loss were found to predict response to immune checkpoint inhibitors.^8^

What has not been well established is the prognostic role of individual MMR genes and *BRAF* mutations, as well as other molecular and clinical factors, in patients with MMR-D mCRC. The primary objective of this study is to identify prognostic factors in MMR-D/MSI-H mCRC in a large patient cohort with a real-world database.

## Methods

Patients with stage IV colon or rectal cancer with MMR-D (defined as loss of MLH1, PMS2, MSH2, or MSH6), determined by immunohistochemistry, or MSI-H, determined by PCR of tumor samples, were selected from the nationwide de-identified, electronic health record (EHR)-derived Flatiron Health database and included in this study. The Flatiron Health database is a longitudinal database, comprising de-identified patient-level structured and unstructured data, curated via technology-enabled abstraction.^9,10^ During the study period, the de-identified data originated from approximately 280 US cancer clinics (~800 sites of care). The majority of patients in the database originate from community oncology settings; relative community/academic proportions vary depending on the study cohort.

*BRAF* and *RAS* mutation status, primary tumor site, and exposure to immunotherapy were noted. ECOG performance status was determined on initial evaluation. The presence of *BRAF* mutations was confirmed by various methods including next-generation sequencing, polymerase chain reaction, and immunohistochemistry, and was stratified by age, gender, *RAS* status, and tumor site. Overall survival (OS) was determined from date of metastatic disease to date of death or last visit date and stratified based on age, *BRAF* status, *RAS* status, type of MMR gene loss, tumor site, and ECOG score. The loss of MLH1 and PMS2 was classified as MLH1 loss, and loss of MSH2 and MSH6 was classified as MSH2 loss, given the functional dependence of MLH1 with PMS2 and MSH2 with MSH6.

Chi-square test was used to examine the association between the *BRAF* mutation and clinical/molecular markers, and the Cox regression model was used for univariate and multivariate analysis. All analyses were performed utilizing SAS version 9.4 (SAS Institute Inc, Cary, NC), and R (Version 4.0.2). Institutional Review Board approval of the study protocol was obtained before study conduct and included a waiver of informed consent.

## Results

A total of 1101 patients with MMR-D/MSI-H mCRC diagnosed from January 1, 2013 to November 25, 2020 were included from the Flatiron Health database. Most patients were older than 50 years (79.9%), Caucasian (75%), and had ECOG 0-1 (83.4%) (Table 1). Of patients with known specific MMR gene loss (*n* = 833), 687 (82.5%) had MLH1 and/or PMS2 loss, while 146 (17.5%) had MSH2 and/or MSH6 loss. Among patients with known *BRAF* status (*n* = 803), 44.3% (*n* = 356) had a *BRAF* mutation and 55.7% (*n* = 447) were *BRAF* wildtype (Table 1). Of patients with *BRAF* mutation, 301 patients had *BRAF* V600E mutation, 18 had a non-*BRAF* V600E mutation, and 27 had an unknown *BRAF* mutation type, and 10 had more than one *BRAF* mutation.

**Table 1.** Baseline patient demographics and clinical/molecular characteristics.

Variable	Level	*N* = 1101	%
Age at diagnosis	≤50	223	20.3
	>50	878	79.7
Gender	Female	593	53.9
	Male	507	46.1
	Missing	1	-
Race	Black or African American	94	9.4
	Other Race	155	15.6
	White	747	75.0
	Missing	105	-
ECOG	0	411	46.9
	1	320	36.5
	2	114	13.0
	3	29	3.3
	4	2	0.2
	Missing	225	-
MMR gene loss	MLH1	607	72.9
	MSH2	93	11.2
	MSH6	53	6.4
	PMS2	80	9.6
	Missing	268	-
MMR gene loss	MLH1+PMS2	687	82.5
	MSH2+MSH6	146	17.5
	Missing	268	-
*BRAF*	*BRAF* mutant	356	44.3
	*BRAF* wild	447	55.7
	Missing	298	-
*RAS*	*RAS* mutant	244	28.9
	*RAS* wild	599	71.1
	Missing	258	-
Tumor Site	Colon	1009	92.5
	Rectum	82	7.5
	Missing	10	-
Immunotherapy	No	750	68.1
	Yes	351	31.9
Age at diagnosis	Median	67	
	Minimum	18	
	Maximum	85	
	Std Dev	14.49	
	Missing	0	

The presence of *BRAF* mutation was more common in age >50 versus ≤50 (52% vs 14.1%, *P* < .001), females versus males (54.8% vs 31.7%, *P* < .001), *RAS* WT versus *RAS* mut (52.8% vs 8.8%, *P* < .001), and colon versus rectum (46.9% vs 14.8%, *P < .*01; Table 2). MLH1/PMS2 loss, compared with MSH2/MSH6 loss, was more common in age >50 versus ≤50 (86.9% vs 64.4%, *P* < .001), females (87.5% vs 76.8%, *P* < .001), *RAS* WT (86.6% vs 64.9%, *P* < .001), and colon (84.6% vs 52.8%, *P* < .001; Table 2).

**Table 2.** Clinical characteristics based on *BRAF* mutation status and MMR genes.

Covariate and level	*BRAF* mutation status			Affected MMR genes		
	Not present	Present	*P* value	MLH1+PMS2	MSH2+MSH6	*P* value
	*n* = 447	*n* = 356		*n* = 687		
					*n* = 146	
Age, years						
≤50	140 (85.9%)	23 (14.1%)	<.001	105 (64.4%)	58 (35.6%)	<.001
>50	307 (48%)	333 (52%)		582 (86.9%)	88 (13.1%)	
Gender						
Female	200 (45.2%)	242 (54.8%)	<.001	386 (87.5%)	55 (12.5%)	<.001
Male	246 (68.3%)	114 (31.7%)		301 (76.8%)	91 (23.2%)	
*RAS* Status						
Mutated	176 (91.2%)	17 (8.8%)	<.001	109 (64.9%)	59 (35.1%)	<.001
Wildtype	241 (47.2%)	270 (52.8%)		387 (86.6%)	60 (13.4%)	
Tumor Site						
Colon	394 (53.1%)	348 (46.9%)	<.01	655 (84.6%)	119 (15.4%)	<.001
Rectum	46 (85.2%)	8 (14.8%)		28 (52.8%)	25 (47.2%)	

**Figure 1.**
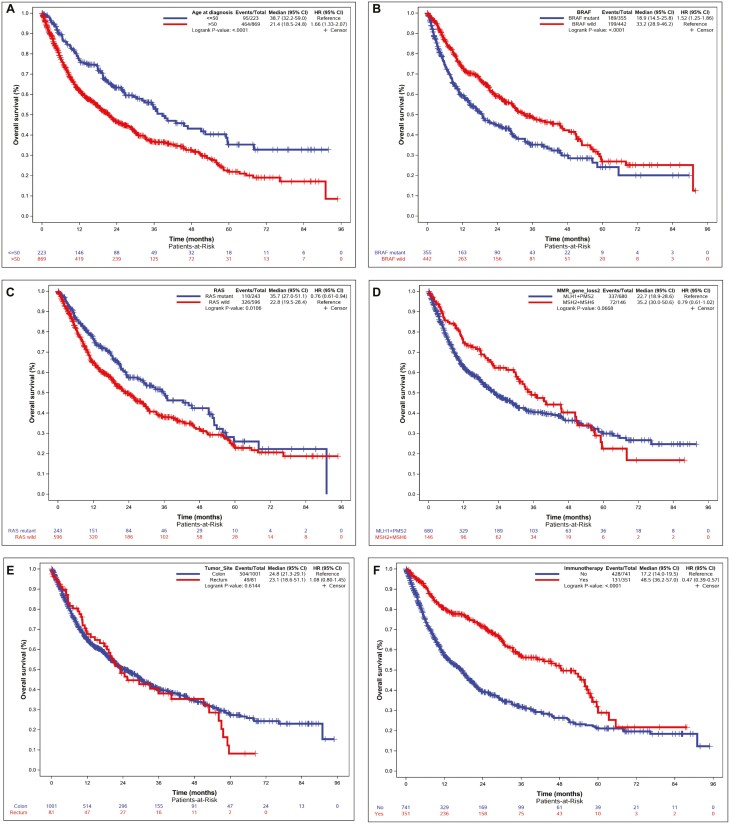
Kaplan–Meier progression-free survival curves based on (**A**) age, (**B**) *BRAF* mutation status, (**C**) *RAS* mutation status, (**D**) MMR gene loss (MLH1+PMS2 vs MSH2+MSH6), (**E**) tumor site, and (**F**) exposure to immunotherapy.

Patients with *BRAF* mutation also had worse survival outcomes compared to patients with wild-type *BRAF* with overall survival of 18.9 versus 33.2 months (HR 1.52, 95% CI: 1.25-1.86, *P* < .001; Fig. 1). A subset analysis of patients with *BRAF* V600E mutation also showed worse OS compared with wild-type *BRAF* with overall survival of 17.3 versus 33.2 months (HR 1.59, 95% CI: 1.29-1.96, *P* = <.001; Supplementary Fig. 1). Patients aged >50 were found to have worse OS compared with patients age ≤50 with a median survival time of 21.4 months versus 38.7 months (HR 1.66, 95% CI: 1.33-2.07, *P* < .001; Fig. 1B). Patients with a *RAS* mutation had improved OS compared with patients with wild-type *RAS* at 35.7 versus 22.8 months (HR 0.76, 95% CI: 0.61-0.94, *P* = .011; Fig. 1C; Tables 3 and 4).

**Table 3.** Overall survival analysis based on various clinical/molecular characteristics.

Variable name	Level	Median survival time (months) with 95% CI
Age at diagnosis	≤50	38.7 (30.5-52.4)
	>50	21.4 (18.4-24.5)
Immunotherapy	Yes	48.5 (36.2-56.5)
	No	17.2 (14.0-19.2)
*BRAF*	*BRAF* mutant	18.9 (14.4-25.4)
	*BRAF* wild	33.2 (28.9-46.2)
*RAS*	*RAS* mutant	35.7 (27.0-51.1)
	*RAS* wild	22.8 (19.5-28.2)
MMR gene loss	MLH1	22.8 (18.6-28.8)
	MSH2	34.0 (29.3-59.5)
	MSH6	37.1 (19.1-50.3)
	PMS2	22.7 (12.5-30.4)
MMR gene loss	MLH1+PMS2	22.7 (18.8-28.4)
	MSH2+MSH6	35.2 (29.9-50.3)
Tumor Site	Rectum	23.1 (17.7-40.2)
	Colon	24.8 (21.2-28.9)

**Table 4.** Univariate Cox regression analysis with reported HRs.

			OS		
Covariate	Level	*N*	HR (95% CI)	HR	Log-rank
				*P*-value	*P*-value
Age at diagnosis	>50	869	1.66 (1.33-2.07)	**<.001**	**<.001**
	≤50	223	-	**-**	
Gender	Male	502	1.13 (0.96-1.34)	.146	.147
	Female	589	-	**-**	
Race	Black or African American	92	0.98 (0.73-1.31)	.874	.787
	Other Race	154	1.09 (0.85-1.39)	.519	
	White	742	-	**-**	
ECOG performance status	2-4	142	2.23 (1.76-2.83)	**<.001**	**<.001**
	0-1	728	-	**-**	
MMR gene loss	MSH2	93	0.72 (0.52-1.00)	**.047**	.166
	MSH6	53	0.95 (0.65-1.40)	.812	
	PMS2	79	1.13 (0.83-1.54)	.451	
	MLH1	601	-	**-**	
MMR gene loss	MSH2+MSH6	146	0.79 (0.61-1.02)	.068	.067
	MLH1+PMS2	680	-	**-**	
*BRAF*	*BRAF* mutant	355	1.52 (1.25-1.86)	**<.001**	**<.001**
	*BRAF* wild	442	-	**-**	
*RAS*	*RAS* mutant	243	0.76 (0.61-0.94)	**.011**	**.011**
	*RAS* wild	596	-	**-**	
Tumor Site	Rectum	81	1.08 (0.80-1.45)	.615	.614
	Colon	1001	-	**-**	
Immunotherapy	Yes	351	0.47 (0.39-0.57)	**<.001**	**<.001**
	No	741	-	**-**	
Age at diagnosis		1092	1.02 (1.02-1.03)	**<.001**	
Duration on immunotherapy (months)		351	0.89 (0.87-0.92)	**<.001**	

Bold values are statistically significant.

Abbreviations: CI, confidence interval; HR, hazard ratio; OS, overall survival.

There was no significant difference in overall survival between individual MMR genes (*P* = .166); however, when MSH2/MSH6 were grouped and compared with MLH1/PMS2 mutations, a trend toward improved overall survival was noted with a median survival time of 35.2 versus 22.7 months (HR 0.79, 95% CI: 0.61-1.02, *P = .*067; Fig. 1D). No significant difference in OS was seen based on primary tumor site, colon versus rectum at 23.1 versus 24.8 months (HR 1.08, 95% CI: 0.80-1.45, *P = .*614; Fig. 1E). Treatment with immunotherapy did lead to an improved median survival time of 48.5 versus 17.2 months (HR 0.47, 95% CI: 0.39-0.57, *P* < .001; Fig. 1F; Tables 3 and 4).

On multivariate Cox regression analysis, which included all significant variables identified in univariate analysis, increased age, ECOG 2-4 (HR 1.87, 95% CI: 1.38-2.54, *P* < .0001) and *BRAF* mutation (HR 1.41, 95% CI: 1.08-1.85, *P* = .0121) remained to be associated with worse survival outcomes. Treatment with immunotherapy (HR 0.49, 95% CI 0.38-0.64, *P* < .0001) continued to show improved survival in multivariate analysis, while *RAS* mutation status lost its significance (Table 5).

**Table 5.** Multivariate Cox regression analysis with reported HRs.

Variable	Level	HR (95% CI)	*P* value
Age at diagnosis		1.01 (1, 1.02)	.0254
ECOG performance status	2-4	1.87 (1.38, 2.54)	<.0001
	0-1	-	-
*BRAF*	*BRAF* mutant	1.41 (1.08, 1.85)	.0121
	*BRAF* wild	-	-
Immunotherapy	Yes	0.49 (0.38, 0.64)	<.0001
	No	-	-

Abbreviations: CI, confidence interval; HR, hazard ratio.

## Discussion

Significant heterogeneity among patients with MMR-D CRC exists; therefore, to understand this disease entity better, we sought to determine the clinical and molecular features that play a prognostic role in MMR-D CRC. In our study, we identified that *BRAF* mutations and age >50 to be associated with worse survival outcomes for patients with MMR-D mCRC.

*BRAF* V600E mutation has been well established as a poor prognostic marker in MSS CRC. This particular point mutation maintains an active *BRAF* kinase, leading to constitutive activation of the MAPK pathway and upregulation of the cell cycle, propagating carcinogenesis.^11^ In MMR-D tumors, *BRAF* mutations are more common with an incidence of 34.6% compared with MSS tumors at 6.8%.^12^ This may be due to *BRAF*’s association with the high-level CpG island methylator phenotype (CIMP) and MLH1 promoter methylation.^13^

For early-stage MMR-D CRC, *BRAF* mutation has been established as a poor prognostic factor, based on analysis of survival after disease recurrence from the ACCENT database. This database included 7 trials with patients with stage III CRC treated with adjuvant therapy with a total of 271 patients with MMR-D CRC. Patients with the *BRAF* V600E mutation with MMR-D (*n* = 91) were noted to have worse survival compared with patients with *BRAF* wild-type MMR-D (*n* = 180) (HR 2.65, 95% CI 1.67-4.21, *P* < .0001).^14^

Our study is the first and largest study, to our knowledge, to identify *BRAF* mutations’ association with worse overall survival (18.9 months vs 33.2 months, HR 1.41, *P* = .01) for MMR-D/MSI-H mCRC in a large patient cohort. While this mutation can be associated with other factors that can contribute to worse survival, including older age, we demonstrated that the presence of *BRAF* mutations is still associated with worse survival outcomes on multivariate analysis. Previously, one pooled analysis of 4 studies of MMR-D mCRC patients did not show differences in PFS or OS based on *BRAF* mutation status with PFS 6.1 versus 6.3 months (HR 1.07, 95% CI 0.67-1.70, *P* = 1.0) and OS 11.7 versus 15 months (HR 1.51, 95% CI: 0.93-2.46, *P* = .155).^12^ However, this analysis involved a sample size of just 153 patients and only 53 patients with *BRAF* V600E mutation. In addition to being prognostic, the *BRAF* mutation may be predictive of response to immunotherapy: MMR-D patients with *BRAF* V600E mutation treated with immunotherapy have been found to have worse 1-year and 2-year PFS compared with those who were *BRAF* wildtype (40% vs 73.3% and 26.7 vs 73.3%, respectively, *P* < .001) when treated with immunotherapy.^8^

Age >50 was another adverse prognostic factor identified for MMR-D mCRC in our study. For patients with stage II-IV MMR-D CRC, Oh et al. found that age >65 was associated with worse survival (HR 3.191, 95% CI 1.27-8.021, *P* = .014).^15^ Worse outcomes with older age may be expected given a higher chance of comorbidities, inability to tolerate aggressive treatment regimens, and decreased overall life expectancy. Notably, the presence of *BRAF* mutations, which were found as an adverse prognostic factor in our study, was more common in patients age >50. However, after adjustment in multivariate analysis, which included *BRAF* mutations, age >50 remained a poor prognostic factor, suggesting additional clinical and biological factors may have an impact on survival outcomes of late-onset MMR-D mCRC. It is important to note that, while providers are more likely to undertreat older patients with CRC regardless of underlying comorbidities, the decision to offer various treatment options to patients should not be made solely on age.^16^

An ECOG performance score of 2-4 was associated with adverse survival outcomes, compared with ECOG 0-1 in our cohort of patients. ECOG performance score ≥2 has been well established as an adverse prognostic marker for all patients with mCRC and non-metastatic CRC regardless of MMR status.^17,18^ In the MMR-D population, ECOG score remains an important determinant of prognosis. Also, as expected, patients treated with immunotherapy in our study had significantly improved overall survival compared to those who did not receive immunotherapy (48.5 vs 17.2 months, HR 0.47, 95% CI: 0.39-0.57, *P* < .001).

The association of *RAS* mutations with poor clinical outcomes in early-stage MMR-D CRC has been established; however, studies of its role in advanced disease are limited. One study of patients with stage I-IV CRC found that KRAS mutated/MMR-D CRC had the shortest OS while KRAS WT/MMR-D CRC had the longest OS.^19^ Specifically, in early-stage MMR-D CRC, KRAS status has not been established to have prognostic value; this may be due to the smaller sample sizes and difficulty with reaching statistical significance.^20,21^ Our study evaluating over 1000 mCRC patients failed to identify this association; conversely, we discovered an association between wild-type *RAS* status and adverse survival outcome, although this did not persist on multivariate analysis. Notably, *RAS* mutations and *BRAF* mutations are almost mutually exclusive, and worse outcomes observed in univariate analysis for *RAS* wild-type patients could be related to the predominant presence of *BRAF* mutations in the *RAS* wild-type cohort. It is also important to note that, while *BRAF* V600E is known to be a founder mutation in MMR-D CRC, *RAS* mutations are not driver alterations in MMR-D CRC and occasionally occur as a result of frameshift mutations; their molecular and clinical significance remains to be seen.

We also evaluated differences in survival outcomes for MMR-D mCRC patients based on the type of protein loss leading to the MMR deficiency. Patients were grouped into MLH1/PMS2 vs. MSH2/MSH6 categories, given the functional dependence of these proteins on each other. Loss of MLH1/PMS2 protein demonstrated a trend toward worse overall survival compared with MSH2/MSH6 loss (*P* = .067). Of note, MLH1 loss is often seen in association with *BRAF* mutation, which has a poor prognosis, as mentioned previously. Also, MLH1/PMS2 loss is associated with a low tumor mutational burden, which is also a negative prognostic factor.^22^ Other studies evaluating the prognostic value of individual MMR proteins or MMR protein subtypes are limited; therefore, additional studies are needed to verify our findings. Of note, the type of individual MMR protein loss has been shown to confer differences in response to immunotherapy in MMR-D CRC. Patients with MSH2/MSH6 loss have had greater 1-year and 2-year PFS compared with those with MLH1/PMS2 loss (84.2% vs 57.8% and 78.2% vs 54.2%, respectively, *P* < .001) when treated with immunotherapy.^8^

Our study is limited by its retrospective nature; however, the large sample size allowed for statistically significant results that were not seen with smaller-sized studies discussed above. Also, our dataset did not include other potential biomarkers such as sidedness of cancer, tumor mutational burden, and sites of metastasis, which may also have prognostic implications. Additionally, the mutation type for those with KRAS/NRAS mutations, as well as whether or not surgery was part of patients’ treatment, were not specified in the Flatiron Health database. Furthermore, prospective studies are needed to confirm our findings.

Currently, MMR-D mCRC is viewed as one disease entity with generalized treatment guidelines. However, multiple clinical and molecular features were found in our study to confer differences in survival outcomes for patients with MMR-D mCRC, including *BRAF* mutation status, age, and ECOG score. A trend toward differences in overall survival was also noted based on the type of MMR protein loss. These findings highlight the heterogeneity of MMR-D CRC as well as the importance of incorporating these factors into understanding the pathophysiology of this disease.

## Conclusion

In conclusion, our study demonstrated that *BRAF* mutations and age >50 are associated with inferior survival outcomes for patients with MMR-D mCRC. *RAS* mutations and specific MMR alterations do not seem to be associated with survival outcomes. As we gain a better understanding of the interplay of these prognostic factors, our approach to managing MMR-D CRC can become more personalized and hopefully lead to improved survival for patients.

## Supplementary Material

oyab055_suppl_Supplementary_Figure

## Data Availability

The data underlying this article will be shared on reasonable request to the corresponding author.

## References

[1] Arnold M, Sierra MS, Laversanne M, Soerjomataram I, Jemal A, Bray F. Global patterns and trends in colorectal cancer incidence and mortality. Gut. 2017;66(4):683-691. 26818619 10.1136/gutjnl-2015-310912

[2] Rahib L, Wehner MR, Matrisian LM, Nead KT. Estimated projection of US cancer incidence and death to 2040. JAMA Netw Open. 2021;4(4):e214708. 33825840 10.1001/jamanetworkopen.2021.4708 PMC8027914

[3] Liu B, Nicolaides NC, Markowitz S, et al. Mismatch repair gene defects in sporadic colorectal cancers with microsatellite instability. Nat Genet. 1995;9(1):48-55. 7704024 10.1038/ng0195-48

[4] Liu B, Parsons R, Papadopoulos N, et al. Analysis of mismatch repair genes in hereditary non-polyposis colorectal cancer patients. Nat Med. 1996;2(2):169-174. 8574961 10.1038/nm0296-169

[5] Liu GC, Liu RY, Yan JP, et al. The heterogeneity between lynch-associated and sporadic MMR deficiency in colorectal cancers. J Natl Cancer Inst. 2018;110(9):975-984. 29471527 10.1093/jnci/djy004

[6] Kloor M, von Knebel Doeberitz M. The immune biology of microsatellite-unstable cancer. Trends Cancer. 2016;2(3):121-133. 28741532 10.1016/j.trecan.2016.02.004

[7] Andre T, Shiu K-K, Kim TW, et al. Pembrolizumab versus chemotherapy for microsatellite instability-high/mismatch repair deficient metastatic colorectal cancer: the phase 3 KEYNOTE-177 study. J Clin Oncol. 2020;38(18_suppl):LBA4-LBA. 10.1016/S1470-2045(22)00197-8 PMC9533375 35427471

[8] Sahin IH, Goyal S, Pumpalova Y, et al. Mismatch Repair (MMR) Gene Alteration and BRAF V600E mutation are potential predictive biomarkers of immune checkpoint inhibitors in MMR-deficient colorectal cancer. Oncologist. 2021;26(8):668-675. 33631043 10.1002/onco.13741 PMC8342606

[9] Birnbaum BNN, Seidl-Rathkopf K, Agrawal M, et al. Model-Assisted Cohort Selection with Bias Analysis for Generating Large-Scale Cohorts from the EHR for Oncology Research. Cornell University; 2020.

[10] Ma X, Long L, Moon S, et al. Comparison of population characteristics in real-world clinical oncology databases in the US: flatiron health, SEER, and NPCR. medRxiv. 2020. 10.1101/2020.03.16.20037143

[11] Safaee Ardekani G, Jafarnejad SM, Tan L, Saeedi A, Li G. The prognostic value of BRAF mutation in colorectal cancer and melanoma: a systematic review and meta-analysis. PLoS One. 2012;7(10):e47054. 23056577 10.1371/journal.pone.0047054 PMC3467229

[12] Venderbosch S, Nagtegaal ID, Maughan TS, et al. Mismatch repair status and BRAF mutation status in metastatic colorectal cancer patients: a pooled analysis of the CAIRO, CAIRO2, COIN, and FOCUS studies. Clin Cancer Res. 2014;20(20):5322-5330. 25139339 10.1158/1078-0432.CCR-14-0332 PMC4201568

[13] Lochhead P, Kuchiba A, Imamura Y, et al. Microsatellite instability and BRAF mutation testing in colorectal cancer prognostication. J Natl Cancer Inst. 2013;105(15):1151-1156. 23878352 10.1093/jnci/djt173 PMC3735463

[14] Taieb J, Shi Q, Pederson L, et al. Prognosis of microsatellite instability and/or mismatch repair deficiency stage III colon cancer patients after disease recurrence following adjuvant treatment: results of an ACCENT pooled analysis of seven studies. Ann Oncol. 2019;30(9):1466-1471. 31268130 10.1093/annonc/mdz208 PMC7360150

[15] Oh BY, Huh JW, Park YA, et al. Prognostic factors in sporadic colon cancer with high-level microsatellite instability. Surgery. 2016;159(5):1372-1381. 26775578 10.1016/j.surg.2015.11.028

[16] Yamano T, Yamauchi S, Kimura K, et al; Japanese Study Group for Postoperative Follow-up of Colorectal Cancer. Influence of age and comorbidity on prognosis and application of adjuvant chemotherapy in elderly Japanese patients with colorectal cancer: A retrospective multicentre study. Eur J Cancer. 2017;81:90-101. 28622612 10.1016/j.ejca.2017.05.024

[17] Sargent DJ, Köhne CH, Sanoff HK, et al. Pooled safety and efficacy analysis examining the effect of performance status on outcomes in nine first-line treatment trials using individual data from patients with metastatic colorectal cancer. J Clin Oncol. 2009;27(12):1948-1955. 19255311 10.1200/JCO.2008.20.2879 PMC2669760

[18] Lu CS, Chang PY, Chen YG, Chen JH, Wu YY, Ho CL. Stage III colon cancer: the individualized strategy of adjuvant chemotherapy for aged under and over 70. PLoS One. 2015;10(9):e0138632. 26382962 10.1371/journal.pone.0138632 PMC4575165

[19] Hu J, Yan WY, Xie L, et al. Coexistence of MSI with KRAS mutation is associated with worse prognosis in colorectal cancer. Medicine (Baltimore). 2016;95(50):e5649. 27977612 10.1097/MD.0000000000005649 PMC5268058

[20] Zhang X, Ran W, Wu J, et al. Deficient mismatch repair and RAS mutation in colorectal carcinoma patients: a retrospective study in Eastern China. PeerJ. 2018;6:e4341. 29423347 10.7717/peerj.4341 PMC5804321

[21] de Cuba EM, Snaebjornsson P, Heideman DA, et al. Prognostic value of BRAF and KRAS mutation status in stage II and III microsatellite instable colon cancers. Int J Cancer. 2016;138(5):1139-1145. 26376292 10.1002/ijc.29855

[22] Salem ME, Bodor JN, Puccini A, et al. Relationship between MLH1, PMS2, MSH2 and MSH6 gene-specific alterations and tumor mutational burden in 1057 microsatellite instability-high solid tumors. Int J Cancer. 2020;147(10):2948-2956. 32449172 10.1002/ijc.33115 PMC7530095

