# Correction to: *BRAF* Mutations Are Associated with Poor Survival Outcomes in Advanced-stage Mismatch Repair-deficient/Microsatellite High Colorectal Cancer

**DOI:** 10.1093/oncolo/oyac098

**Published:** 2022-05-21

This is a correction to Tan E, Whiting J, Xie H, et al. *BRAF* mutations are associated with poor survival outcomes in advanced-stage mismatch repair-deficient/microsatellite high colorectal cancer. *The Oncologist*. 2022;27(3):191–197. https://doi.org/10.1093/oncolo/oyab055

In the original online publication of this article, Figure 1C appeared in error, as a duplicate of Figure 1B. The error was corrected online before print publication.

