# Supplementary material for: BRAF Mutations Are Associated with Poor Survival Outcomes in Advanced-stage Mismatch Repair-deficient/Microsatellite High Colorectal Cancer
Source: Oncologist. 2022 Feb 9;27(3):191–7. doi: 10.1093/oncolo/oyab055 (PMC8914499; doi:10.1093/oncolo/oyab055)
Supplement: oyab055_suppl_Supplementary_Figure [file oyab055_suppl_supplementary_figure.docx]

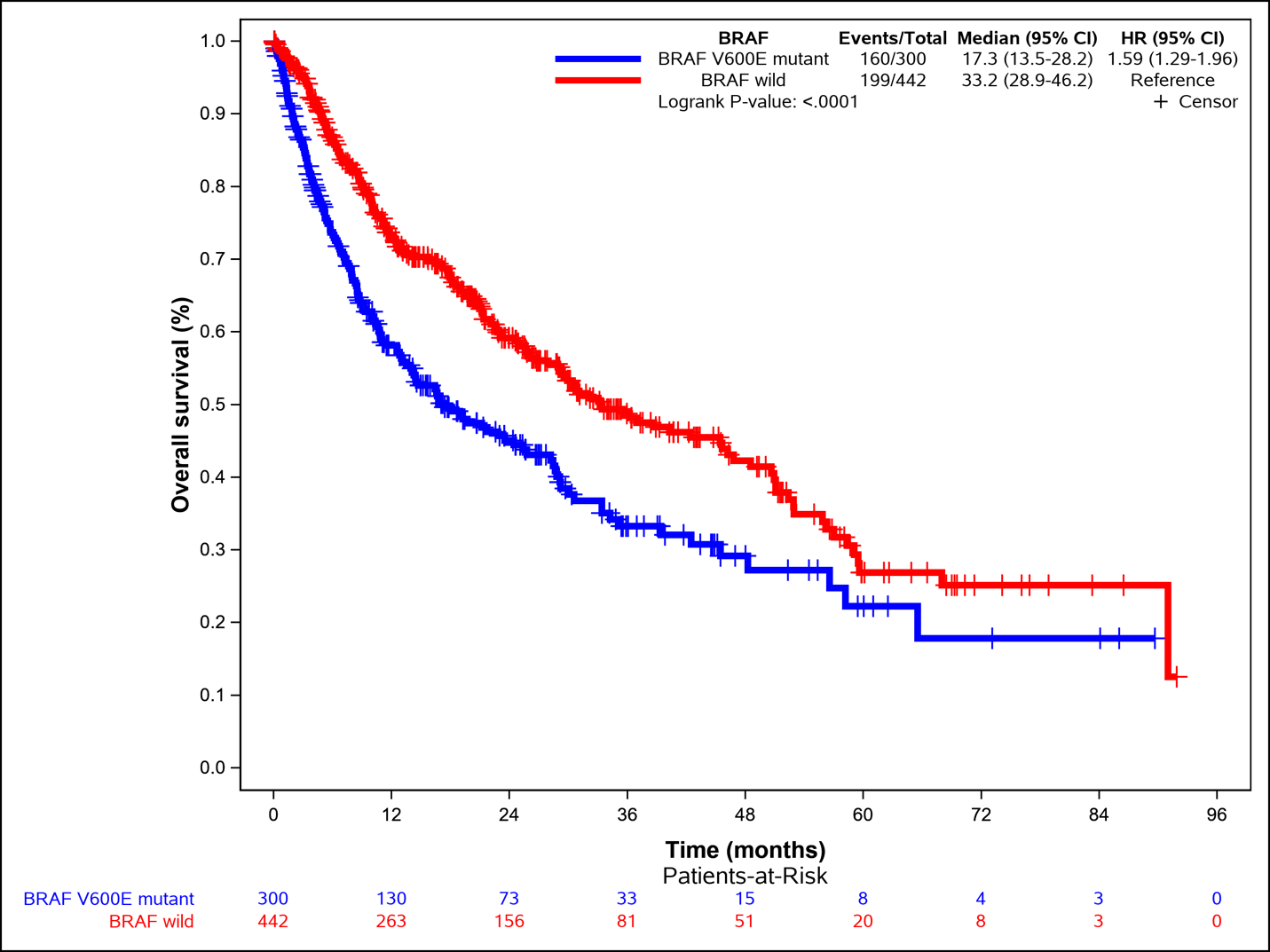


Supplementary Figure 1. Kaplan Meier survival curve of BRAF V600E mutant patients vs. BRAF wildtype patients.
